# Young Doctors Program-Colorado: An Elective Elementary School Program to Improve Health Literacy, Utilization of the Healthcare System, and Osteopathic Awareness

**DOI:** 10.1007/s40670-024-02150-5

**Published:** 2024-09-24

**Authors:** Samantha Sposet, Riley Fabich, Matthew McEchron

**Affiliations:** Rocky Vista University College of Osteopathic Medicine, 8401 S. Chambers Rd., Englewood, CO 80112 USA

**Keywords:** Elementary, Children, Health literacy, Osteopathic medicine, Health care, Career, Medical education, Leadership

## Abstract

The Young Doctors Program (YDP) is a “mini medical school” series for fifth-grade elementary children. The YDP was designed and led by medical students in the Academic Medicine and Leadership Track at the Rocky Vista University College of Osteopathic Medicine in Colorado. The curriculum of the program was designed around the State of Colorado Learning Standards with the purpose of educating students about emergencies and common ailments in five body systems (cardiovascular, respiratory, gastrointestinal, musculoskeletal, and renal) while also providing exposure to healthcare as a profession. YDP provides children with insights into the healthcare system, an introduction to osteopathic medicine, and it enhances community outreach. This article provides a framework for the semester-long YDP curriculum as well as our final event which synthesizes learned information using standardized patients. We describe successes including community outreach and healthcare exposure in the elementary school, and challenges initiating connections with local schools and funding. Future goals include expanding outreach to other fifth-grade schools as well as growth to different age levels. Authors plan to pursue IRB approval for research to examine the benefits and efficacy of the YDP.

## Introduction

The Young Doctors Program (YDP) at Rocky Vista University College of Osteopathic Medicine (RVUCOM) is an elective health education program provided to fifth-grade elementary children in the Parker, Colorado region. The curriculum and lessons are developed and delivered by RVUCOM medical students with faculty supervision. The elementary school–aged children acquire knowledge of human body systems, common ailments, and osteopathic medicine, and they learn about proper utilization of the healthcare system. The program also serves to grow relationships between RVUCOM and the surrounding community. The YDP also gives pre-clinical medical students the opportunity to develop leadership and teaching skills. As the program grows, we intend to expand the program to multiple schools and grade levels.

The YDP was designed with multiple goals in mind. One of the primary goals of the program is to promote health literacy in elementary school–aged children. Other work has shown that health literacy programs can have an impact on children’s ability to make healthy and informed choices [1–3]. Previous research has shown that health intervention programs can have a significant impact on health outcomes as well [4, 5]. The YDP health literacy curriculum outlined in this article also includes information about making informed healthcare decisions and provides an introduction about how the healthcare system should be utilized.

Another goal of the YDP curriculum is to expose elementary-aged students to careers in medicine. There have been a number of programs that have been developed to expose elementary-aged children to various healthcare careers [6–8]. Yavuz (2022) suggests that early career awareness in elementary-aged children encourages student reflection on personal strengths and career interests, and it serves to help students understand educational requirements for certain careers [9]. Data suggests that early career education can improve student academic achievement, and the elementary-aged children with average ability seem to profit the most in their academic achievement [10]. The YDP sought to provide career education regarding practice differences between osteopathic and allopathic medicine, physician’s assistants, and various nursing career tracks. The goal was not to emphasize any career pathway, but to distinguish how different providers function and interact in the healthcare setting.

Another unique goal of YDP is to increase osteopathic awareness in the community and to strengthen community relationships with RVUCOM. Examination of the literature suggests that few if any programs have been developed that introduce osteopathic principles or awareness in elementary school–aged children. Additionally, parents and teachers who attend the standardized patient encounter and graduation ceremony are also able to gain exposure to osteopathic medicine by listening to group discussions and having conversations with current osteopathic medical students. The YDP provides a fun and engaging environment for elementary children to improve health literacy and increase osteopathic awareness.

The final objective of YDP is to develop teaching and leadership skills in our medical students. Reports have shown that teaching opportunities are limited at most medical schools [11]. A number of authors have suggested that medical students should receive formal teaching instruction because the role of a physician in clinics and hospitals is very similar to that of a teacher [12]. The YDP offered students an opportunity to engage in smaller or larger teaching roles depending on the medical student’s interest. Related to teaching and leadership, medical students participating in YDP are able to work closely with several elementary schools in the region. This allowed medical students to expand their communication and leadership training beyond the walls of their medical school.

The YDP program was originally developed by Dr. David Park, D.O. on the RVUCOM Utah campus as part of the Academic Medicine and Leadership (AML) track. The original YDP program in Utah was modified for the AML track at RVUCOM in Colorado. The AML track on both campuses provides an elective experience that runs longitudinally across all 4 years of medical school and focuses on leadership skills in various areas of academic medicine. This manuscript focuses specifically on the YDP program for the RVUCOM Colorado campus.

### YDP Application and Curriculum Overview

We developed the YDP health literacy curriculum around the State of Colorado’s Academic Standards for Comprehensive Health for fifth graders [13]. We targeted the areas of Physical and Personal Wellness, Social and Emotional Wellness, and Prevention and Risk Management. These are outlined in Standards 2, 3, and 4 of the Colorado Academic Standards Online shown in Table 1.

**Table 1:** Health relevant Colorado Academic Standards for fifth-grade children

**Comprehensive Health—Standard 2. Physical and Personal Wellness**
• Demonstrate the ability to make good decisions about healthy eating behaviors
• Explain structure, function, and major parts of the human reproductive system
• Describe physical, social, and emotional changes at puberty
• Demonstrate interpersonal communication skills needed to discuss personal health problems to establish and maintain personal health and wellness
• Comprehend concepts and identify strategies to prevent the transmission of the disease

**Comprehensive Health—Standard 3. Social and Emotional Wellness**
• Analyze internal and external factors that influence mental and emotional health

**Comprehensive Health—Standard 4. Prevention and Risk Management**
• Demonstrate the ability to make good decisions about drug use: marijuana, illegal drugs, prescription drugs, alcohol, and tobacco
• Demonstrate pro-social behaviors that reduce the likelihood of physical fighting, violence, and bullying
• Demonstrate basic first aid and safety procedures

Physical and Personal Wellness (Standard 2 in Table 1) was targeted using a session emphasizing preventative care where topics such as healthy eating, balanced meals, drinking water, and exercising were discussed. Lessons stressed a balance between nutrition and fitness. Social and Emotional Wellness (Standard 3) was targeted by discussing the importance of having social connections with peers and healthy habits such as joining recreational sports leagues. Prevention and Risk Management (Standard 4) was targeted by discussing the risks of tobacco use on cardiovascular and pulmonary health in age-appropriate terms. As the Young Doctors work through standardized patients in the final event, they are encouraged to think about how social factors contribute to health outcomes. Standard 4 was further targeted by teaching students how to properly utilize the healthcare system. For example, lessons discussed when a patient should seek emergency care vs. when they might benefit from seeing a pediatrician/primary care provider. Special care was taken throughout the course to encourage fifth-grade elementary school–aged children to always bring medical issues to the attention of adults at home or school.

The YDP curriculum was structured to provide students with six introductory system sessions followed by a field trip to RVUCOM which included a series of simulated patient encounters and a graduation ceremony. A program overview was presented to school principals and science teachers at local elementary schools. Schools that chose to participate in the program provided the sponsorship of at least one fifth-grade teacher who would serve as the point of contact for the medical student program directors. At the beginning of the term, the fifth-grade children were presented with a program overview, take-home permission slip, and application. Children were given 1 week to complete the permission slip and application which consisted of the simple open-ended question “Why are you interested in joining the Young Doctors Program?”. The program was able to accommodate every fifth-grade student who applied to the program each semester, receiving 26 applications out of approximately 70 total children the first semester and 35 applications out of approximately 85 total children the second semester.

After developing the curriculum, the authors recruited fellow students in the RVUCOM AML Track to teach each of the six lessons to the fifth-grade children at their respective elementary schools during the lunch period. Session topics included cardiovascular, respiratory, musculoskeletal, gastrointestinal, renal systems, and an introduction to osteopathic principles and the basics of taking care of your body. The learning objectives for these topics are outlined in Table 2. Medical students in the AML track volunteered in pairs to teach these sessions using the created curriculum. During each session, the medical-student-teachers were accompanied by one or both of the medical student program directors. This allowed the fifth-grade students to interact with several medical students throughout the program while ensuring that the fifth-grade children, teachers, and staff saw a familiar face and point of contact each session.

**Table 2:** Body systems and key learning objectives

	**Cardiovascular**	**Respiratory**	**Musculoskeletal**
Anatomy	• Understand the four chambers and valves of the heart • Trace the path of blood through the heart	• Identify the anatomical structures that make up the respiratory system • Understand the path of air in exhalation and inhalation	• Learn what a joint is and use the basic terminology of moving a joint • Identify major muscles of the body • Identify major bones in the body
Function	• Describe the function of the cardiovascular system	• Describe the function of the respiratory system	• Describe the function of the musculoskeletal system
Introduction to diagnostic tools	• Recognize an ECG and name key information that can be learned from one	• Use a stethoscope to hear breath sounds	• Understand the use of an x-ray in diagnosing a fracture
Clinical correlations	• Define a myocardial infarction. Recognize signs and symptoms	• Compare and contrast asthma and an upper respiratory infection	• Compare and contrast a ligament sprain with a fractured bone • Identify emergency signs of a fracture
Activity	• Draw the heart as a “house” with four rooms representing the four chambers. Label the doors and windows representing the valves and trace the path of blood through the house • Practice taking your own pulse	• Practice listening to breath sounds with a stethoscope. Students lined up and were coached on using a stethoscope to listen to lungs by the medical students	• “Simon says” with bones and muscles learned. Example: “Simon says touch your tibia” or “Simon says flex your hamstring”
	**Gastrointestinal**	**Renal**	**Osteopathic introduction and taking care of our bodies**
Anatomy	• Trace the pathway of food through the digestive tract • Recognize anatomical structures in each of the four abdominal quadrants	• Identify the renal cortex, medulla, renal artery and vein, and ureter • Name the functional unit of the kidney • Point to the location of your kidneys	• Understand the relationship between structure and function in our bodies
Function	• Describe the function of the gastrointestinal system	• Describe the function of the renal system	• Describe the different impacts of exercise, diet, and smoking on our bodies
Introduction to diagnostic tools	• Understand the use of ultrasound to look at the appendix	• Name some information that can be learned from a urinalysis	• Understand why an osteopathic physician may use their hands to screen for a somatic dysfunction
Clinical correlations	• Name the signs and symptoms of appendicitis • Identify the treatment of appendicitis	• Identify the signs and symptoms of kidney stones and compare these with previous diagnoses discussed	• Describe a somatic dysfunction and how it may impact someone
Activity	• Kahoot! To test knowledge	• Jeopardy to test knowledge	• Practice combining different body movements to understand how our bodies are one unit

**Fig. 1:**
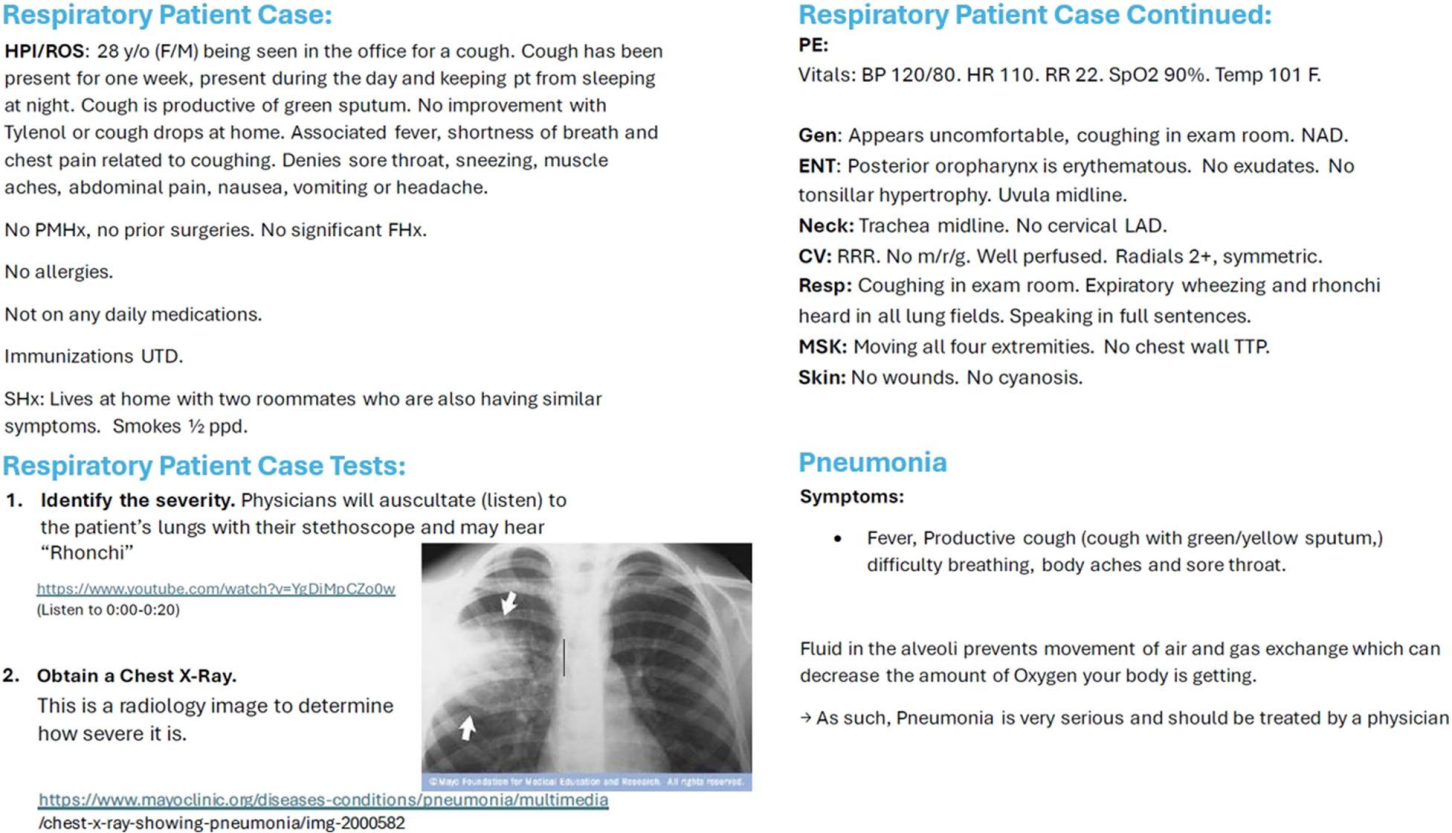
Partial standardized patient case example—pneumonia

Following these classroom sessions, the fifth-grade children took a field trip to the RVUCOM medical school where they participated in small group patient encounters with medical students acting as their patients. Small groups of about five children worked as a team to engage in five total patient cases. During the patient cases, each group was assigned to a medical student who facilitated the patient visit, exam skills, and group discussion while another medical student acted as the patient. These five mini cases were designed to integrate information from earlier sessions with new concepts in the setting of a patient encounter. A partial case example is shown in Fig. 1. In this example case a patient presented with respiratory symptoms. The medical students facilitated a discussion about chest x-rays and how they may provide useful information in a case like this. The medical students guided the fifth-grade children through very basic details on a chest x-ray including identifying and counting ribs, identifying the heart outline, and noticing any areas of opacity or “cloudiness” in the lung fields that may indicate pneumonia. Student facilitators used this information as a guide during discussions with the children in small groups. The field trip culminated in a graduation ceremony for the Young Doctors. Parents and families were invited to attend the graduation and a short reception.

## YDP Objectives

The purpose of this program is fourfold: build a foundation of health literacy among local fifth-grade children, increase RVUCOM’s interaction with the community while increasing an overall awareness of the osteopathic profession, provide elementary school–aged children with exposure to healthcare as a career choice, and develop the teaching and leadership skills of medical students.

### Health Literacy

**Fig. 2:**
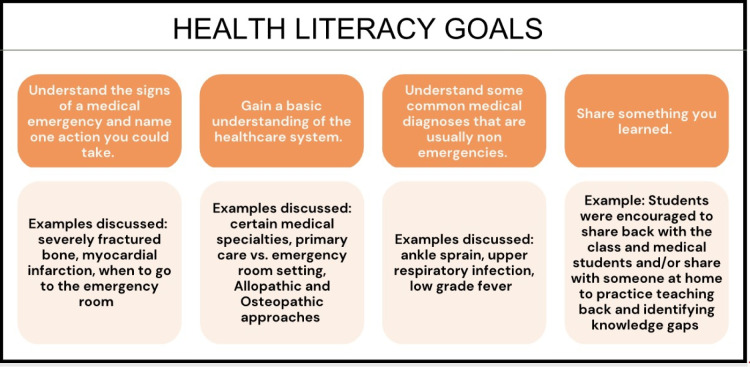
Health literacy goals of the Young Doctors Program with examples

YDP was developed in part to provide early exposure to health-related material with the aim of increasing children’s health literacy. Sessions were developed with the goal of increasing elementary-aged children’s understanding of the healthcare system and basic and common medical diagnoses. In addition, we sought to introduce the discussion of when to seek medical care and what constitutes a medical emergency. Students were encouraged to think critically about what health meant to them personally and to make choices that aligned with their idea of a healthy lifestyle. Students were always encouraged to immediately share medical issues with adults at home and at school. Health literacy goals of the program and examples are listed in Fig. 2.

### Healthcare Career Exposure

Exposure to careers in medicine and healthcare was one of the main objectives of YDP. Many of the fifth-grade children expressed an interest in careers in science and medicine in their applications to the program. Most of these students had experience with the field of healthcare as a patient, but we hoped to encourage exploration of healthcare as a career. Previous work about career development in elementary aged children suggests that early career education can improve student academic achievement, and elementary aged children with average ability seem to profit the most in their academic achievement [10]. The YDP allowed fifth-grade elementary school children an opportunity to interact with medical students who are at an early stage of the medical education journey. The YDP lessons provide career education regarding practice differences between osteopathic and allopathic medicine, physician’s assistants, and various nursing career tracks. The goal was not to emphasize any career pathway, but to distinguish how different providers function and interact in the healthcare setting.

### Exposure to Osteopathic Medicine and Strengthening Community Relationships

YDP also sought to establish and maintain ties between RVUCOM and the surrounding local community. Established in 2006, Rocky Vista is a relatively young school in a rapidly growing suburb of Denver. The YDP-Colorado served as an introduction not only to Rocky Vista University but also to osteopathic medicine for many of the teachers, children, and families who participated. YDP increased exposure to the osteopathic profession and articulated the similarities and differences between osteopathic and allopathic physicians. The program included a dedicated Osteopathic session that exposed the fifth-grade children to osteopathic manipulative therapy (OMT) and created a foundation of understanding when OMT may be safe and appropriate. More information on this specific session can be found in Table 2.

### Develop Teaching Skills in Medical Students and Strengthen Community Connections

The final objective of YDP was to enhance the teaching skills of medical students and provide an opportunity to practice community engagement. Participation in the program allowed medical students to interact with the community and practice teaching in a fun, low-stakes environment. This is important because most medical students will encounter some sort of teaching role in the various stages of their medical education [11]. In addition, patient education is an essential function of a physician. Whether teaching a medical student as a resident later in their career, or teaching a patient about their new diagnosis, it is essential for medical students to be exposed to teaching opportunities early in training. There are limited opportunities in the pre-clinical curriculum to practice the teaching skills required to achieve effective patient education. Several studies have described the importance of providing patient education opportunities to medical students [14]. YDP sought to provide these opportunities by allowing our medical students to teach various sessions to the fifth-grade children.

## Osteopathic Exposure

The YDP curriculum included a session titled, “Osteopathic Medicine & Taking Care of Your Body,” which introduced osteopathic medicine to the fifth-grade children and teachers. In this session, the fifth-grade children were taught the four tenants of osteopathic medicine and introduced to the definition of somatic dysfunction through objectives as outlined in Table 2. The medical students led discussions about how the body operates as a unit and how to consider the body, mind, and spirit, when determining treatments for a patient. Fifth-grade children were asked to brainstorm different animals with abilities humans do not have and think about how the structure of those animals might be related to their distinct functions. Additionally, the children were introduced to Fryette’s laws of spinal mechanics, which are a foundation of osteopathic medicine [15]. Students participated in an activity where they were asked to consider different spine movements like side bending, rotation, and flexion or extension and think about how moving one part of the spine affects the rest of the body. Finally, AML facilitators discussed viscerosomatic dysfunctions and lymphatic treatments by reviewing upper respiratory infection symptoms related to the sessions about respiratory function. To help understand the importance of structure and function and the goal of osteopathic medicine, students were verbally introduced to the Galbreath technique and lymphatic drainage [15]. Facilitators discussed the importance of antibiotics when needed, but introduced the idea that by treating somatic dysfunctions, the body may be able to heal itself without additional medications. This is supported in a study by Degenhardt et al. who showed that the progression of recurrent otitis media can be altered by osteopathic treatments [16]. During this discussion, the children were introduced to treatment approaches of both allopathic and osteopathic medicine. It was discussed that both share the goal of treating patients and providing the best care possible. During the standardized cases, AML facilitators helped elementary school children brainstorm diagnoses and treatment plans which incorporated the osteopathic principle of holistic care that aims to treat the whole patient. Throughout this session, parents were invited to speak with the AML facilitators, medical students, and RVUCOM faculty. Faculty and students facilitated a discussion about osteopathic medicine and answered questions about how osteopathy differs from allopathic medicine and chiropractic treatments.

Examination of the literature suggests that the introduction of osteopathic medicine to grade school children is a novel idea. By introducing children to osteopathic medicine, this program gives individuals the information to eventually choose a provider that is best for their care, whether allopathic or osteopathic. For students who are inspired to practice medicine, it may also allow them to make an informed decision about pursuing osteopathic vs. allopathic medicine. Anecdotally, authors believe that many pre-medicine students applying to medical school do not have exposure to osteopathic medicine and struggle to define the practice when compared to allopathic medicine. There is limited research about osteopathic awareness in pre-medicine students. However, a recent study showed that only 31% of MD students were aware of OMT as a musculoskeletal treatment modality [17]. Their study also showed that MD students have an interest in taking an elective in OMT [17]. YDP may help to educate students from an early age about osteopathic medicine. YDP may also help to reduce biases related to osteopathic medicine and increase understanding of the differences between the osteopathic and allopathic professions.

## Successes

YDP was successful at building relationships between RVUCOM and local elementary schools in Parker, CO. Students from two elementary schools participated in the YDP. Relationships were built with over 50 elementary school students throughout the semester, along with their teachers and parents during our standardized patient and graduation event. Parents had the opportunity to watch their children during simulated standardized patient cases in cardiovascular, respiratory, gastrointestinal, renal, and neurological systems. These patient cases allowed students to synthesize learned information throughout the course and become familiar with different patient cases using a whole person approach. It was clear that students retained information from previous course sessions.

Anecdotally, parents, teachers, and students expressed enthusiasm for the lunchtime program and the authors were told about family dinner discussions that included YDP content. Parents expressed gratitude for the early exposure to healthcare practices and careers. Elementary school teachers, parents, and students expressed interest in continuing the YDP beyond the fifth-grade level. One future goal of YDP is to expand the program to other schools and to include higher grade levels with synchronous curricula. Plans for this expansion have already begun.

The YDP program provided an opportunity for medical students to grow as leaders and future physicians. The program allowed medical students to develop curricula, practice teaching skills, lead peers, and manage financial and logistic aspects of the program. It is important to note that the American Academy of Family Physicians states that patients should be educated at a sixth grade or lower level and ideally education should include pictures and illustrations [11]. To this end, medical students in the AML Track were engaged in a similar parallel practice through the YDP. The YDP created numerous opportunities for AML students to engage with their community. YDP also helped medical students develop leadership and academic skills, one of the main goals of the AML track.

## Challenges

One of the early challenges for YDP was curriculum design as it required careful planning, research, and selection of content. The authors used Colorado’s fifth-grade standards and their personal experience with similar age groups, to design each session to be highly interactive at an age-appropriate level. The curriculum was also shaped in part by a pre-existing YDP on the RVUCOM’s Utah campus with the notable difference that the YDP-Utah requires the medical student volunteers to create the curriculum for the session they sign up to facilitate. The YDP-Colorado program is unique in that the curriculum has been standardized across sessions, so that session content could build on previous content, and the sessions would remain age-appropriate regardless of the experience of the medical student facilitators. In addition, this change addressed the challenge of recruiting busy medical students as volunteers. Presenting a pre-designed curriculum required less time from the medical student volunteers as opposed to creating and presenting their own content.

Another challenge was identifying elementary schools that may be interested and willing to participate in the program. The program was designed to require minimal effort from teachers at the school, which was reassuring for teachers and important for establishing connections with potential schools. The only expectations from the teacher liaison was to provide classroom space, collect applications, and communicate information about the field trip with parents. Outcome data collection will be a challenge moving forward. The local school district we are working with is limiting IRB applications until August 2025. We plan to submit an IRB to administer pre- and post- program surveys to better evaluate outcomes.

The final challenge was securing and allocating resources for YDP. The AML Track included funding to pay for food and drink for a short reception after the graduation ceremony, t-shirts for the fifth graders to keep, and white coats for the fifth-grade students to be reused for each class of Young Doctors. This budget was also used for printing handouts for the sessions, graduation certificates, signage, and applications. Future resources may include take home workbooks for the fifth-grade students. This would allow information and progress to be shared with parents and family members. Transportation for the field trip was handled by the schools and each school elected to use parent transportation.

## Conclusions and Future Goals

Supplemental health literacy programs for elementary aged children are not widely developed or published in the United States. YDP provides an innovative approach for improving health literacy, healthcare system knowledge, and osteopathic awareness in young children. Enthusiasm for the YDP program from teachers, YDP students, and medical students has been positive within the first 2 years of operations.

This program’s early successes have been encouraging, and plans are in place to expand YDP to other schools. The standardized learning objectives, teaching modules, and standardized patient cases will provide ease of expansion to additional elementary schools and grade levels.

As we move in this direction, we will continue to refine the learning objectives and lessons to reinforce Colorado’s state requirements. This could allow synchronous curricula across schools and grade levels. We envision that higher grade level learning objectives will cover body systems in greater depth and expand on more complicated pathophysiology and treatment plans. While educating students about health through a mind, body, and spirit approach, future lessons may involve topics such as fitness, diet, sexual health, preventative medicine, and emotional wellness. Eventually, the goal for this program is to allow for an elective, supplemental learning experience for elementary aged children through high school. We anticipate that this will provide excellent exposure for children considering a healthcare career.

As the program grows, we will open volunteer teaching positions to the entire RVUCOM student body. With more volunteers, it will be possible to run simultaneous programs at different schools and ensure that there are enough medical students to meet the demands of the elementary school children. However, one of the limitations of the program has been budget and resources. We plan to investigate additional funding options for future programs.

Finally, we plan to pursue IRB approval to examine YDP students’ knowledge before and after participation in the program. We also plan to obtain survey data from students, teachers, and family members. These efforts will provide valuable information about the impact of YDP among participants and family members.
